# The art of the float

**DOI:** 10.1167/jov.23.8.13

**Published:** 2023-08-16

**Authors:** Emily A. Cooper, Roberto Casati, Hany Farid, Patrick Cavanagh

**Affiliations:** 1 University of California, Berkeley, Berkeley, CA, USA; 2 Institut Jean Nicod, ENS, EHESS, CNRS, PSL University, Paris, France; 3 Glendon College and CVR, York University, Toronto, Ontario, Canada

**Keywords:** vision, shadows, depth, art

## Abstract

For more than 2000 years, artists have exploited cast shadows to influence how objects appear to be positioned in a scene. A *contact cast shadow* can anchor an object to the ground and a *detached cast shadow* can make an object appear to float. However, there is a period of approximately 1000 years when there were virtually no cast shadows in art. How were states of contact versus floating depicted by artists without cast shadows? Here, we survey various techniques used by artists to anchor relative position with and without cast shadows. We then conduct experimental tests of the hypothesized surface attraction principles that underlie these techniques. In the absence of cast shadows, an object (a wooden box) was often seen as resting on a surface as long as that surface offered information about ground orientation and support (a tiled floor). When the ground surface was ambiguous and cloud-like (1/f noise), the box was more likely to be seen to float. The presence of cast shadows made the box appear to contact the ground whether it was well-defined or ambiguous. Both shadows and surface support also increased the accuracy with which participants detected when the box was tilted up from the ground. These results indicate that artists long ago discovered the important power of support relationships to anchor objects to surfaces in the absence of shadows.

## Introduction

Where there is light, there are shadows. Shadows represent both a challenge and a convenience for vision. To parse a scene successfully, visual systems must distinguish shadows from objects or material features. At the same time, shadows can signal an object's presence, location, and shape, as well as the relief and illumination level on the surface on which they fall. Because of the realism they offer, shadows have fascinated visual artists: painters, graphic designers, and movie makers. These artistic explorations constitute a rich vein of visual experiments offering more than 2000 years of documented discoveries in the use of cast shadows to anchor objects to the surface below them. Starting around 400 BCE, Greek and then Roman painters mastered many aspects of cast shadow depiction, but strangely, after being used for more than 800 years in paintings and mosaics, cast shadows vanished from documented pictorial art for the following 1,000 years (Gombrich, 1995; Casati & Cavanagh, 2019). Here we ask what painters used to replace the anchoring and floating roles of cast shadows during this period.

What is a cast shadow? Light interacts with objects in many ways. Reflected light from an object's surface, for example, depends on the angle of incidence, creating shading effects that enhance the surface relief of an object. To create a shadow, parts of an object must block light completely from other parts of its own surface (attached or self-shadows) or block the light falling on other surfaces (cast shadows). It is the latter, cast shadows, that are most effective in anchoring objects in the scene or making them appear to float (Gombrich, 1995; Casati & Cavanagh, 2019).

**Figure 1.**
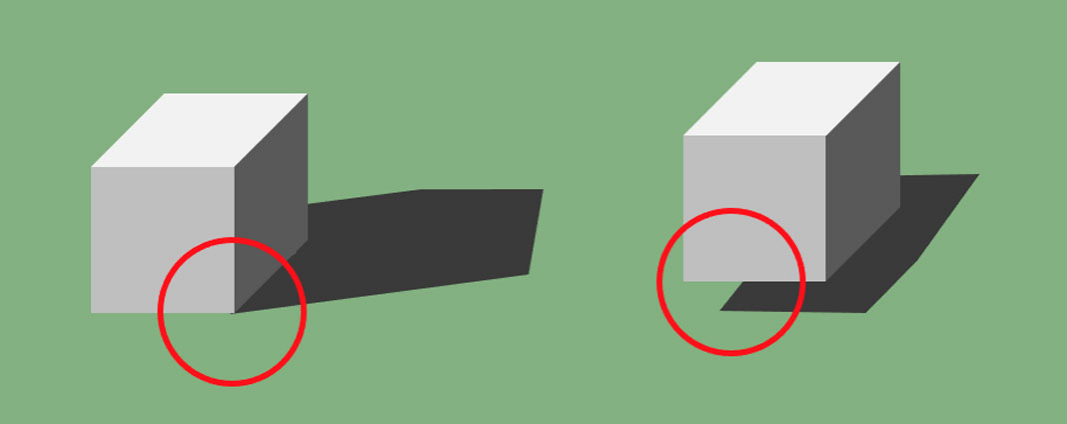
The contact cast shadow on the left anchors the object to the surface it sits on. The shadow must extend from the object, meeting it where the light grazes the object's external boundary typically making a K-junction (red circle) where the three object contours meet the shadow contour. The detached shadow on the right indicates that the object is floating over the surface. If the shadow is partly occluded by the object, it makes a T-junction with the object contour. Image credit: PC.

Cast shadows have characteristic visual features (Mamassian, Knill & Kersten, 1998; Casati, 2004; Casati & Cavanagh, 2019). When an illuminated object sits on a surface, any direct light will cast a shadow of the object onto the surface, on the side opposite the light source extending out from the object. This is a contact cast shadow. Importantly the shadow area on the surface must meet the object's boundary where it is in contact with the surface, typically making a K-junction with the object's contours (Figure 1, left). When the object is floating without contacting the surface, the object and its shadow may be visually separated or, if the object partially occludes its shadow, it forms only T-junctions with it (Figure 1, right). This is a detached cast shadow. The offset of the shadow from the object provides an index of the separation between the object and the surface (Kersten, Knill, Mamassian, & Bülthoff, 1996; Cavanagh, Casati, & Elder, 2021). A cast shadow may be a sharp copy of the object's shape viewed from the light's direction if the light source is compact. For more diffuse light, though, the cast shadow is a more triangular gradient that fades away with distance from the object. We first describe the positioning roles of cast shadows in art and then go on to explore how these positioning roles were filled in the 1,000 years when painters avoided any cast shadows. These techniques, discovered by painters during that time, will lead us to insights about how perception recovers three-dimensional positions from two-dimensional images, which we follow with an experiment to test these hypotheses.

## Cast shadows in art

**Figure 2.**
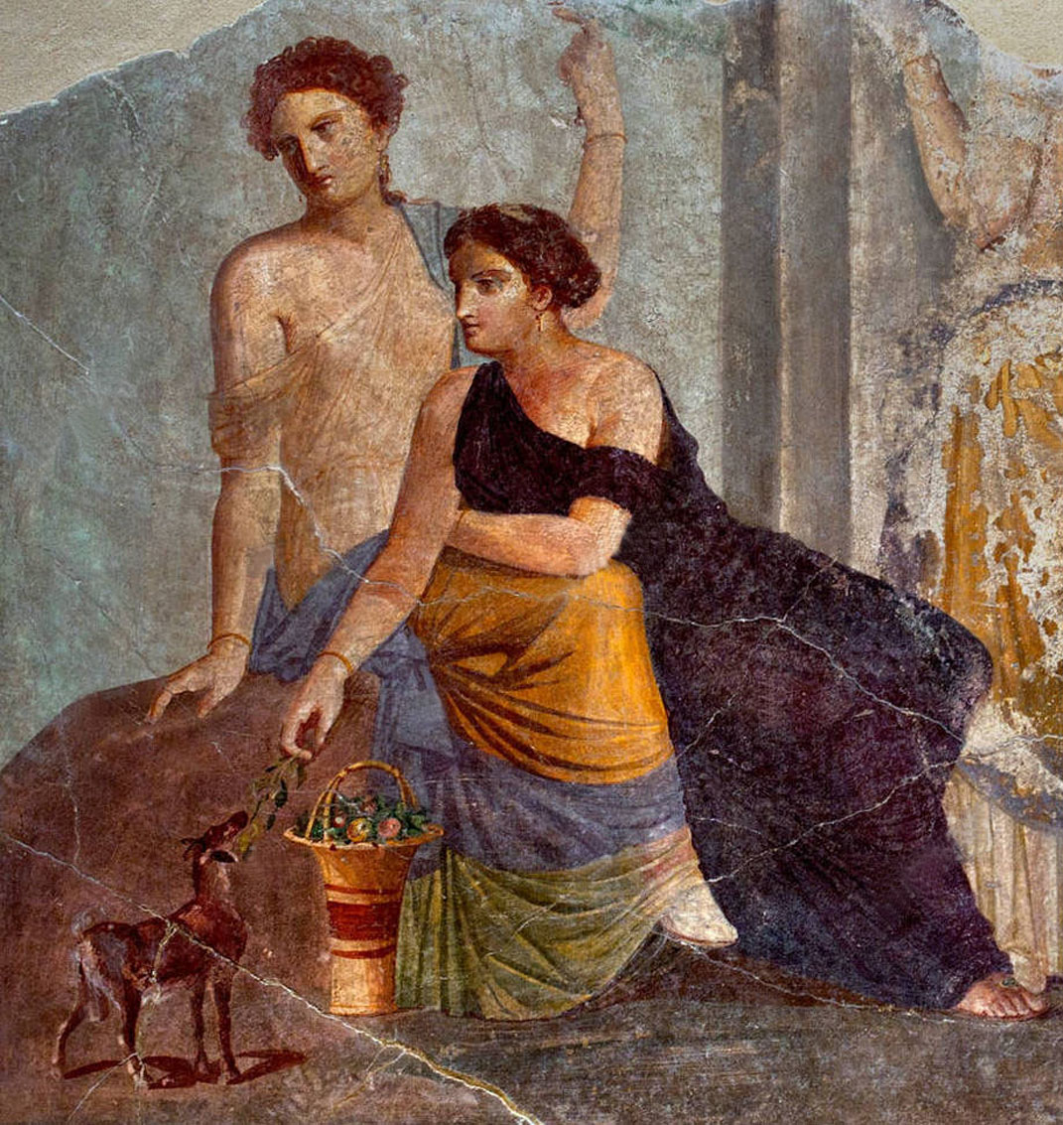
Anchoring cast shadows can be seen under the fawn, indicating that its hooves contact the surface. Fresco, 30 to 50 CE Pompeii, detail from Woman beside a fawn. © 2012 RMN-Grand Palais (musée du Louvre)/Stéphane Maréchalle, https://collections.louvre.fr/en/ark:/53355/cl010283853.

Contact cast shadows are widely used in art to anchor objects to the surfaces beneath them. This anchoring only became important once Greek painters introduced a receding ground plane. Previously, Egyptian painters, for example, used only a one-dimensional, straight line to depict the ground plane with no extension in depth in the image. Objects, animals and people stood on this line to indicate they were resting on the ground. The arrival of the realist painters of the fifth century BCE introduced both the receding ground plane and the cast shadows that anchored objects to it (Pliny the Elder, trans. Bostock & Riley, 1855). These techniques were then widely used in Roman art, as seen in the example fresco in Figure 2 (Gombrich, 1995). In paintings, most shadows originate at a character's feet or an object's base, to signal that the depicted elements are located at some specific spot, anchored on the ground, and are not floating above it (Casati & Cavanagh, 2019). The anchoring function does not require sophisticated shadow representations: a few marks on the ground that suggest a darkening of the area at the contact point are in general enough. This visual appearance corresponds to the typical features of cast shadows in the presence of combined diffuse and directional light. Although the visual system is quite tolerant of unrealistic shadow shapes (Mamassian, 2004; Jacobson & Werner, 2004; Ostrovsky et al, 2005; Nightingale, Wade, Farid, & Watson, 2019), there is a limit; by the end of the Roman era, the anchoring cast shadows became a convention using implausible black marks whose effect was more symbolic than visual.

**Figure 3.**
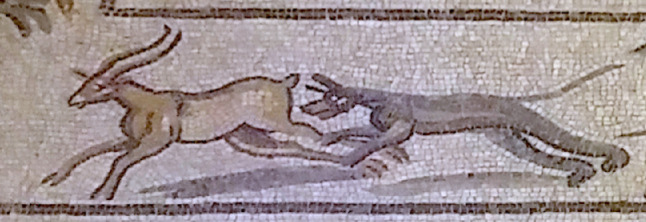
Detached cast shadows depict a separation between the object and the surface on which it falls. Mostly detached shadows are seen under the dog and stag (except for their hind hoof/paw), making them appear to be leaping above the ground. Zeugma Mosaics c. 200 CE, frieze detail from the Birth of Aphrodite. **©** CC-BY-SA-4.0, Wikimedia Commons.

Although anchoring is one function of shadows, another is floating. When a hovering object casts a shadow onto a surface below it (Figure 3), the offset of the shadow is a cue to the distance between the object and the surface (Kersten, Knill, Mamassian, & Bülthoff, 1996; Cavanagh et al., 2021). A hovering object does not always cast a visible shadow. In particular, in diffuse light, the cast shadow may become too faint to be seen when the object is far from the surface below it. Nevertheless, as long as it is visible, it provides depth information when the shadow is linked to the object that casts it.

The shadow's offset from the object that casts it is also affected by the direction of the light, and this direction is often unknown, making the recovery of depth ambiguous. Nevertheless, we seldom experience this ambiguity because the visual system seems to assume a particular fixed direction of light. For example, Kersten et al. (1996) showed that, when a static object casts a moving shadow, viewers see the object move in depth rather than inferring a moving light source with a fixed object location. But what direction of light is assumed? When an object has an offset shadow on a surface behind it, the perceived separation in depth was found to be about twice the shadow's offset, consistent with a light source roughly 30° from the line of sight to the object, on the side opposite to the shadow's offset (Cavanagh et al., 2021). The possibility of an assumed light direction (Boyaci, Doerschner, & Maloney, 2006; Koenderink, Pont, Van Doorn, Kappers, & Todd, 2007) is challenged when faced with several cast shadows that are mutually inconsistent—no possible light source or sources can explain the conflicting directions of the shadows and yet observers immediately see the depths from the shadows (e.g., Ostrovsky, Cavanagh, & Sinha, 2005; Nightingale et al., 2019). Rather than assuming any light source or light field, we have proposed that perceived depth can be directly recovered from shadow offset (Cavanagh, et al., 2021). In any case, these offset shadows, although effective in conveying depth, were used only occasionally in ancient art, and also sparingly in Renaissance and post-Renaissance paintings.

As mentioned elsewhere in this article, for reasons of culture, style, or bad weather, cast shadows disappeared from Western art between about 450 CE and 1410 CE (and were not present in eastern art until modern times) (Gombrich, 1995). Giotto introduced “architectural” shadows in the 1300s by darkening walls under overhanging balconies, for example, but Robert Campin in 1410 was the first to use cast shadows for objects and people, followed by the Limbourg Brothers, Masaccio, Massolino, and a few others by 1425 CE (Gombrich, 1995). Interestingly, shading remained present in art over the 1,000-year period of no cast shadows, indicating that the absence of cast shadows in art was a stylistic choice and not due to a loss of rendering skills. We might think that without any anchoring shadows, the objects and people depicted in artwork during this period might appear to float. Indeed, perhaps floating was the intended effect for the (mostly saintly) individuals depicted in Western art during this period. Actually, this supposition would be wrong. Art from this period does not evoke a strong percept of floating, or a lapse of gravity. It seems that these works are exploiting nonshadow cues to position the objects and people in the scenes. So, which nonshadow image features could be used to anchor objects and which others could make them float? A brief survey of paintings from this period suggests a specific hypothesis: that support properties, or affordances, filled in when cast shadows vanished from Western art. We next examine these support properties and examples from art that exploit them.

**Figure 4.**
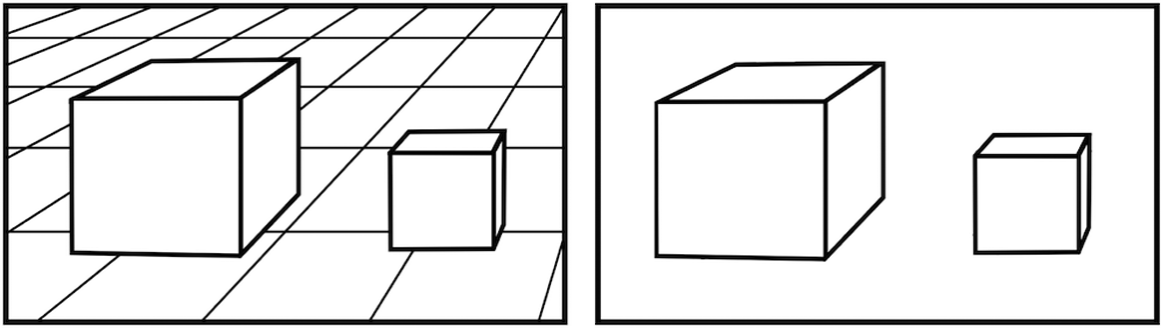
Left, two cubes sit on a tiled ground plane. Right, in the absence of an explicit ground plane, the cubes create a shared plane on which both sit. Adapted from Albert (1999), image credit: PC.

**Figure 5.**
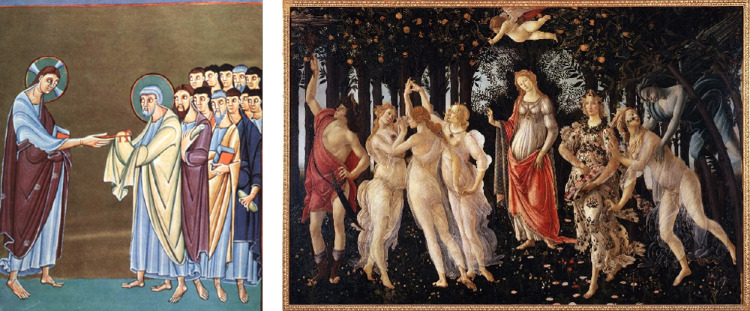
Examples of anchoring support in medieval art, in which the feet are attracted to and create the ground plane. To make a person float, they cannot have any possible contact relation with the ground plane, as with the angel at the top on the right. Left: Pericope book of Henry II, scene: *St. Peter receives the keys*, 11c CE. Right: Sandro Botticelli, *La Primavera*, c. 1480 CE. Public domain, Wikimedia Commons.

By “support properties,” we refer to visual cues that indicate whether an object's base or a person's feet or knees are flush with the ground plane or other surface on which they stand. For example, the tile pattern on a ground plane in Figure 4 on the left provides strong perspective cues for the ground orientation that may be used to create a percept of support and contact with objects. Interestingly, the supporting surface may not be explicitly depicted in the image and may only be generated by the presence of the bases or feet that could be standing on it (Figure 4, right). In Figure 5, we show two artistic examples in which feet serve this function. We are familiar with standard subjective surfaces like the Kanisza triangle that appear in front of partially occluded objects. However, on the right side of Figure 4 and in Figure 5, a subjective ground plane is created underneath the objects that are perceived to rest on it. Albert and Tse (Albert, 1999; Albert & Tse, 2000) first described this power of objects to appear to line up, to become coplanar to create a ground plane on which they sit. We propose that paintings during the shadow-free era used this property of an implied ground plane that attracts the objects and people to its surface, a subjective surface that the people's feet and the objects’ bases create. Importantly, this attraction to the subjective surface explicitly places the object in the three-dimensional scene, despite the ambiguity of the distance between the object and the observer.

**Figure 6.**
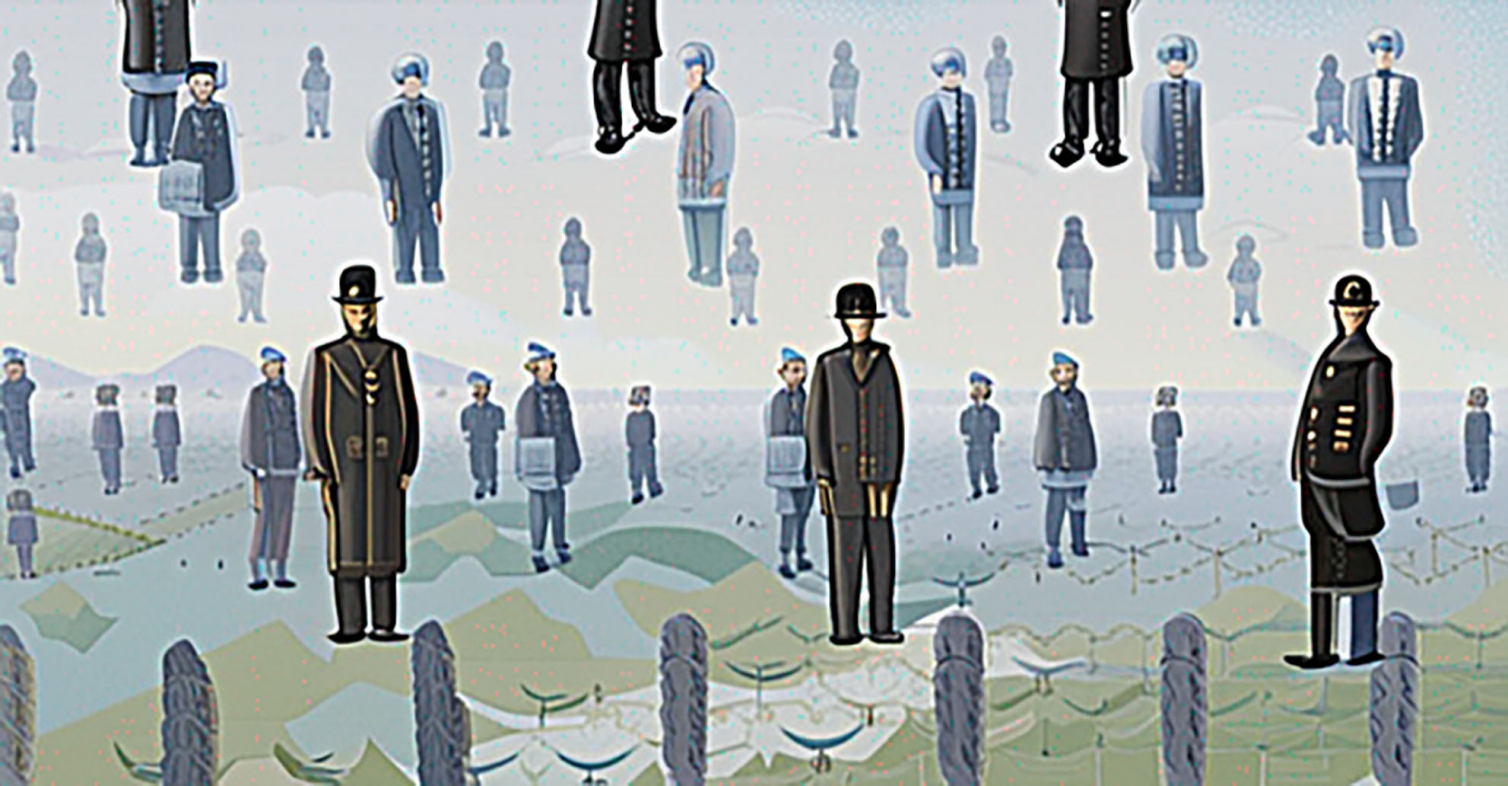
This AI adaptation of Magritte's painting *Golconda*/1953 demonstrates that individuals without possible contact to the ground plane must float, whereas figures with possible contact to the ground mostly seem to stand on it. The version here was adapted by Matyáš Boháček from Magritte's original using Stable Diffusion XL and the prompt “*Golconda by René Magritte, identical black-dressed men standing in lines, bottom, middle, top*.” The original can be viewed here: https://musee-magritte-museum.be/uploads/pages/images/magritte_11719dig_h_1_large@2x.jpg.

If this subjective ground plane pulls objects and people to itself and keeps them from floating, what technique was used to break this attraction when artists wanted to depict objects and people as floating without using detached cast shadows? To break away from the surface attraction, artists perhaps had to show there could be no support—for example, the feet were angled away from the ground. Even better, the image could make it clear that there was no nearby ground to exercise its attraction. Specifically, people or objects surrounded by the sky could only be seen to float, for example, in Figure 5, the angel on the right, and in Figure 6, the individuals with the sky background.

To explore the power of shadows and support properties for placing objects in a scene, we conducted an experiment where we presented an image of a box with and without a contact shadow. The box was above a tiled ground plane in one condition and above an ambiguous ground plane with a pink, cloud-like noise (1/f) texture in the other. We expected the shadow to anchor the box to the ground plane in both cases. However, in the absence of a shadow, we predicted that the box would be attracted more to the tiled ground plane, which offers clear support properties, whereas it would be perceived to float above the noise texture. To test the attractive power of the ground plane, the box was presented in five different poses with increasing angles tilted away from the ground plane.

## Methods

### Perceptual study

#### Participants

Seven adults with normal vision were recruited from a university research subject pool to participate in the study. Study procedures were approved by the institutional review board at Rutgers Camden.

#### Stimuli

**Figure 7.**
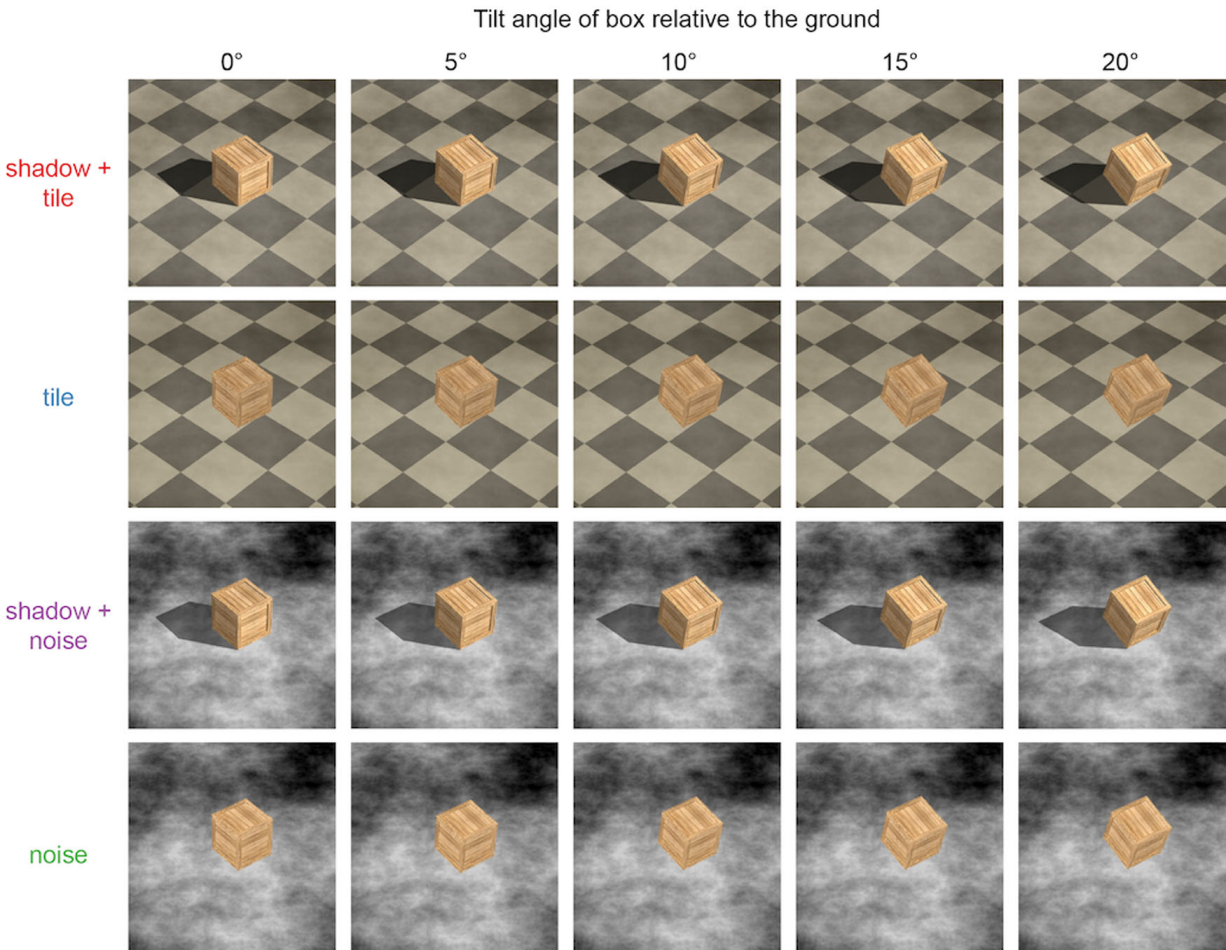
Example images of each stimulus configuration. Rows represent the different stimulus conditions in which ground plane texture and shadows were manipulated. Columns show the five different box poses. The box is flat on the ground in the 0° pose and tilted upward with only one edge on the ground in the other poses. Each image is shown here from the same camera viewpoint for comparison, although the camera viewpoint was randomized in the experiment. The image area has also been cropped for visibility of the box. Image credit: HF and EC.

All stimuli were presented on an iMac at a free viewing distance (approximately 70 cm) in a room with dim ambient illumination. Stimuli were generated using three-dimensional computer graphics in Autodesk Maya. Each stimulus consisted of an image of a wooden box on a ground plane (Figure 7). We rendered the box in a range of poses: it could be sitting flat on the ground plane, or tilted upward such that only one edge was in contact with the ground plane. The box's tilt angle was varied from 0° to 20° in steps of 5°, resulting in five possible poses. We used two different ground plane textures: one with square tiles to provide strong perspective cues about the orientation of the ground that made its support properties clear, and one with a pink (1/f) cloud-like noise texture with substantially weaker linear perspective and little ground location or orientation information.

These scenes were rendered in two different lighting environments: one environment included a combination of both ambient and directional light sources such that the box cast a shadow on the ground plane, and the other was fully ambient such that no shadow was cast. In computer graphics, an additive ambient light term is used to approximate a physically diffuse lighting environment. In the ambient-only lighting environment, the intensity was increased to match the overall luminance level between the two environments. Stimuli were rendered with a square field of view of 27° at a resolution of 1,024 × 1,024 pixels. Thus, there were four stimulus conditions: tile, noise, shadow + tile, and shadow + noise. Altogether, there were five box poses in each of four conditions, generating 20 pose–condition combinations.

We randomly varied the viewpoint of the camera so that there was no one-to-one relationship between the box pose and the two-dimensional projection of the box in the image. For each combination of pose and condition, we rendered the image from two different camera elevations offset by 10° from each other (elevations of 30° and 40° above the ground plane), and nine different camera azimuths in steps of 10°. On each trial, this camera viewpoint was selected at random.

#### Task

Participants were presented with the images one at a time for a duration of two seconds, and were then asked to indicate one of three responses to the question “Is the box is lying flat on floor?”:

• box is not touching ground
• one side of box is flat against ground
• one edge of box is touching ground

We refer to these three responses as floating, flat on the ground, and tilted, respectively.

Participants were presented with, on average, 400 stimuli. Approximately one-half of the stimuli corresponded to a 0° pose (the box was flat on the ground plane). Of the remaining approximately 200 stimuli, one-half corresponded to a 5° pose, one-quarter to a 10° pose, one-eighth to a 15° pose, and one-sixteenth to a 20° pose. We biased the distribution of box poses in this way because normally objects lie flat on the ground, and larger tilt angles are assumed to be increasingly less likely. The total trial number was not deterministic, because this biased distribution was created probabilistically to match the above proportions. The actual number of trials per participant, therefore, ranged from 388 to 407. Trials in each of the stimulus conditions and box poses were randomly interspersed.

#### Analysis

We plotted the mean responses across participants for each condition/pose and analyzed the data using logistic regression to explore how the stimulus properties affected the probability that participants selected each of the three possible responses. Data for each response option were fit separately, resulting in three regression models: one for perceived floating, one for flat on the ground, and one for tilted. Each model included fixed effects for shadow (categorical yes/no), ground texture (categorical as yes/no), and tilt angle (continuous). Individual participants were modelled as random effects, and no interactions were included. Coefficient estimates were exponentiated so as to interpret them as odds ratios. Odds ratios indicate the change in probability of a given response that was associated with changing the value of the fixed effect. Odds ratios close to 1 indicate that variations in the fixed effect were not strongly associated with a change in responses (e.g., the odds of a floating response were very similar whether or not shadows were present). Ratios of greater than 1 indicate an associated increase in the probability of a particular response, and ratios less than 1 indicate a decrease in the probability of a particular response. We expected the presence of shadows and floor tile to be associated with a decrease in the probability of perceived floating and an increase in the probability of perceiving that the box was flat on the ground. A priori, we expected the tilt angle of the box to decrease the probability that the box was perceived flat on the ground and increase the probability that the box was perceived tilted, but we did not have strong hypotheses for how tilt angle might modulate the probability of floating. For each variable in the models, we report the estimated odds ratio, associated model coefficients, 95% confidence interval, *t* statistic, and *p* value.

## Results

**Figure 8.**
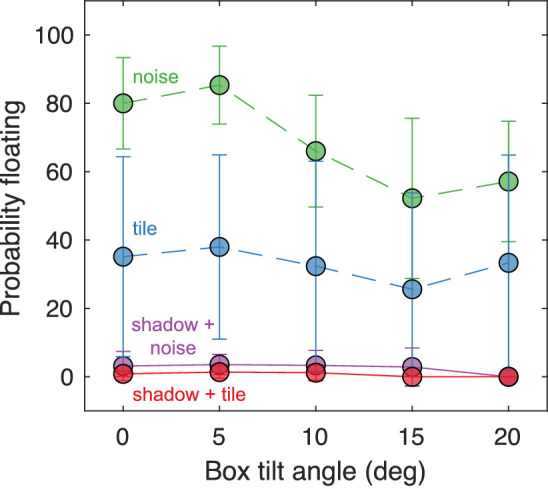
Data associated with the floating response. For each box tilt angle (horizontal axis), the probability that participants responded floating (vertical axis) is plotted separately for each stimulus condition. The averages across participants are plotted as circles and errors bars represent 95% confidence intervals.

We expected that the presence of a shadow would decrease the probability that the box was perceived as floating. The data, and the fitted model, supported this expectation (Figure 8, Table 1). When a shadow was present, participants almost never responded that the box was floating (solid red and purple lines, Figure 8). Floating responses were more prevalent when shadows were absent (dashed green and blue lines, Figure 8). As expected from our hypothesis about support properties, the tile on the ground plane was also associated with reduced floating responses relative to stimuli with a 1/f noise ground plane (blue vs. green lines, red vs. purple lines; Figure 8). Participants were overall most likely to say the box was floating with a noisy ground plane and no shadow (dashed green line; Figure 8) and least likely to say the box was floating with a tiled ground plane and a shadow present (solid red line; Figure 8). There was a slight statistical tendency for increasing tilt angle to be associated with less floating; however, this effect was substantially smaller than the others.

**Table 1.** Variables in the logistic regression model for the floating response.

Variable	Odds ratio	Coefficient estimate	95% confidence interval	*t* statistic	*p* value
Shadow present	0.004	−5.562	−6.055 to −5.070	−22.151	<0.001
Tile present	0.106	−2.243	−2.522 to −1.965	−15.783	<0.001
Tilt angle	0.945	−0.056	−0.077 to −0.035	−5.217	<0.001
Intercept		1.719	0.760 to 2.678	3.513	<0.001

Odds ratios were obtained by exponentiating the coefficient estimates. The regression model had 2,775 degrees of freedom.

**Figure 9.**
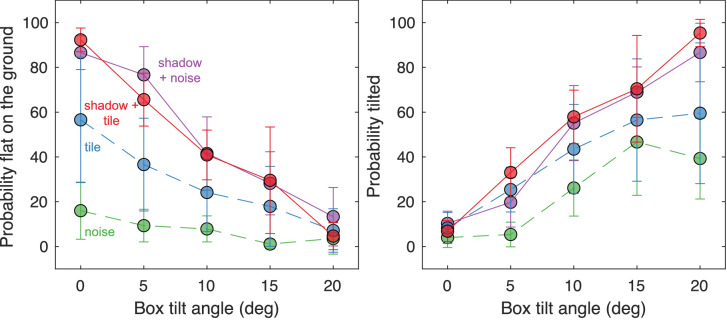
Data associated with the flat on the ground response (left) and the tilted response (right). For each box tilt angle (horizontal axis), the probability that participants made the corresponding response (vertical axis) is plotted separately for each stimulus condition. The averages across participants are plotted as circles and errors bars represent 95% confidence intervals.

We turn next to the responses that the box was flat on the ground (Figure 9 left, Table 2). Consistent with the previous results, we found that both shadows and ground tile were associated with increased odds that participants perceived the box to be flat on the ground. Indeed, when the shadow was present and the box's tilt angle was zero, participants responded correctly that one side was flat on the ground approximately 90% of the time, regardless of the ground texture (solid red and purple lines; Figure 9, left). When the box was presented over the tile without any shadow, it was still strongly perceived as resting flat on the ground (56.6% of the time) when it was, indeed, flat on the ground (tilt angle zero, dashed blue line; Figure 9, left). Across all conditions, as tilt increased, participants were less likely to perceive the box as flat on the ground. This finding makes sense, because in all scenes with a nonzero tilt angle, the box was indeed depicted as angled away from the ground.

**Table 2.** Variables in the logistic regression model for the flat on the ground response.

Variable	Odds ratio	Coefficient estimate	95% confidence interval	*t* statistic	*p* value
Shadow present	11.620	2.453	2.241 to 2.664	22.755	<0.001
Tile present	2.493	0.913	0.719 to 1.108	9.215	<0.001
Tilt angle	0.821	−0.198	−0.217 to −0.178	−19.418	<0.001
Intercept		−0.915	−1.392 to −0.440	−3.774	<0.001

Odds ratios were obtained by exponentiating the coefficient estimates. The regression model had 2,775 degrees of freedom.

For the nonzero tilt poses, as the tilt angle increased participants were increasingly likely to respond that the box was tilted (Figure 9 right, Table 3). Although this pattern was present in all conditions, it was qualitatively the strongest in the presence of shadows (solid red and purple lines; Figure 9, right). When the shadows were gone but the ground plane was tiled, participants were noticeably less accurate (because they were often seeing the cube as floating), but were still able to accurately perceive that the box was tilted 56.5% and 59.5% of the time for the two largest tilt angles, respectively. Without shadows or a tiled ground plane (i.e., the noise condition, dashed green line; Figure 9 right), participants followed the expected trend with increasing tilt, but performance was limited because they more frequently perceived the box as floating (Figure 8).

**Table 3.** Variables in the logistic regression model for the tilted response.

Variable	Odds ratio	Coefficient estimate	95% confidence interval	*t* statistic	*p* value
Shadow present	2.271	0.820	0.607 to 1.033	7.551	<0.001
Tile present	1.659	0.506	0.297 to 0.715	4.755	<0.001
Tilt angle	1.233	0.210	0.192 to 0.228	23.017	<0.001
Intercept		−3.208	−3.611 to −2.805	−15.609	<0.001

Odds ratios were obtained by exponentiating the coefficient estimates. The regression model had 2,775 degrees of freedom.

## Discussion

Cast shadows play an important role in making objects seem to be anchored to the ground or making them float above the ground. We live in a world with gravity, so our experience is of people and objects that are most often not floating. The question we addressed here is how artists make them seem to float. The examples shown in Figures 5 and 6 suggest that the perception of support relationships with a ground plane can fill this role. First, when an object could be in a support relationship, perhaps resting on the ground, it will appear to do so. For example, the feet of people portrayed in front of an untextured field create a ground plane on which all the feet rest (Figure 5). This surface creation is so strong that if artists want to make people appear to float, they may depict them as lacking any possible support relationship to a surface—by bending their legs away from any possible surface beneath them and by placing them where there is no possibility of support, for example, in the sky. These solutions are familiar to us, for example, for astronauts seen floating in and around their space stations, or ballet dancers, gymnasts, and snow boarders in mid jump, and objects tossed overhead. Interestingly, there are not many examples of floating objects in art, perhaps because objects could be lying on the ground in any pose; so, they would only be seen as floating if presented against the sky. Few artists seem to have taken up this challenge.

The experimental results substantiate the power of objects to seek or create a supporting surface. First, in the presence of a cast shadow that extends out from an object (a wooden box), observers almost always reported that the box was in contact with the surface, either resting flush on it or, when tilted away, resting on one edge (Figures 8 and 9). This point was true for the well-defined, tiled surface and for the more ambiguous cloud-like surface textured with noise, as long as the shadow was present.

Without the cast shadow, the box often seemed to float over the noise surface (Figure 8, dashed green line), even though the depiction in all images was always of a box in contact with the surface. This finding is consistent with our proposal that to make an object float without using a detached shadow, artists had to reduce the availability of support surfaces. Our noise surface can appear to be cloud-like, without any solid surface that the box could rest on. In contrast, the tiled surface does appear to offer support. Observers reported that the box either lay flat on the tiled surface or tilted away from it on one edge depending on its angle (Figures 8 and 9).

**Figure 10.**
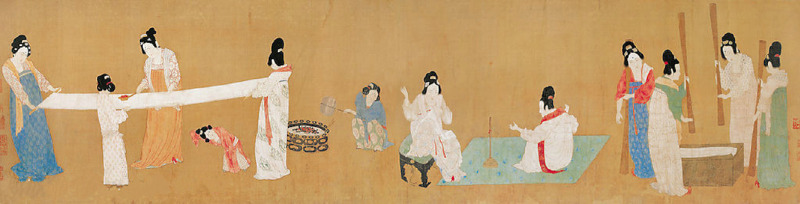
The absence of cast shadows was always the case in eastern art. Receding ground planes were present from ancient times and cast shadows were not used. Instead, the support points of each object create a ground plane that the people and objects stand on. Zhang Xuan, Dao Lian Tu, c. 750 CE. Public domain, Wikimedia Commons.

The outcome for the box above the tiled surface in the absence of the shadow is less clear cut. Our survey of the use of this technique in art suggested that observers would always see the box in contact with the tile surface since it offers well-defined support. However, contact (absence of floating) was reported for only approximately 60% of these trials (Figure 8, blue dashed line). It is possible that the attraction process is stronger with multiple objects as often seen in artworks (Figures 5, 6, and 10), rather than the single object we have tested. We speculate that the addition of more objects in the scenes we tested might further decrease the perception of floating. We had also considered that increasing the angle between the box and the surface would weaken the support relationship and increase reports of floating. However, we did not find this result. Instead, the box more often appears to tilt up on the edge rather than lose contact.

These shortcomings in the expected outcomes suggest that further studies would be helpful. Nevertheless, our findings do confirm that artists could use this support relationship, or surface attraction, to anchor objects and people to the ground plane in the absence of cast shadows. Indeed, this is a property of visual perception that artists, as our first empirical vision scientists, had discovered long ago.
